# The transepicondylar distance is a reliable and easily measured parameter for estimating femoral cartilage surface area using MRI

**DOI:** 10.1002/ksa.12482

**Published:** 2024-09-23

**Authors:** Tayfun Yilmaz, Markus Siegel, Elham Taghizadeh, Andreas Fuchs, Philipp Niemeyer, Hagen Schmal, Kaywan Izadpanah

**Affiliations:** ^1^ Department of Orthopedic Surgery and Traumatology Medical Center—University of Freiburg, Faculty of Medicine University of Freiburg Freiburg im Breisgau Germany; ^2^ Fraunhofer Institute for Digital Medicine MEVIS Bremen Germany; ^3^ Department of Orthopedic Surgery University Hospital Odense Odense C Denmark

**Keywords:** cartilage area, epicondyle distance, knee anatomy, surface corrected defect size, transepicondylar distance

## Abstract

**Purpose:**

Different cartilage repair techniques are widely used to regenerate cartilage, such as autologous chondrocyte implantation (ACI), osteochondral autograft transfer, microfracturing and minced cartilage. Defect size is a key parameter for selecting the best procedure to repair cartilage. However, the defect's size is not related to the patient's total cartilage surface. This is because assessing the femoral cartilage surface area is time‐consuming and therefore unsuitable in the daily clinical routine. It has been proposed that the femur's total cartilage area correlates positively with the transepicondylar distance (TEA).

**Methods:**

The knees of 40 subjects were analysed. Their average age was 30.1 ± 8.6 years. Twenty‐four female and 16 male subjects were examined. Their mean body height was 176.2 ± 8.8 cm. MRI scans were performed via 3‐Tesla MRI. These data were postprocessed and quantified using the browser‐based, customizable SATORI platform (Fraunhofer MEVIS). This software computed the femoral cartilage surface area (FeCA), the patella cartilage surface area (PCA), the TEA and the patella length.

**Results:**

Body height reveals a good correlation (*r *= 0.722, *p* < 0.001) with the distal femur's cartilage area surface. However, regression analysis shows only moderate dependence (*R* ^2^: 0.514). A very good correlation (*r* = 0.830, *p* < 0.001) was observed between the TEA distance and the total cartilage surface area of the distal femur. The regression analysis yields a good value (*R* ^2^: 0.684). The cranio‐caudal length of the patella was chosen as a suitably measurable two‐dimensional parameter for correlation analysis with the patella's total cartilage surface area. Those results yield a poor correlation (*r* = 0.577, *p* < 0.001) between the two parameters, and regression analysis reveals a low value (*R* ^2^: 0.384).

**Conclusion:**

The TEA is a reliable parameter for estimating the femur's cartilage area using MRI. A simple determination of this parameter allows the estimation of the femur's total cartilage area as well as the surface‐corrected defect size (SCDS) in daily routine.

**Level of Evidence:**

Level II

**Abbreviations**
3T: 3 Tesla
ACI: autologous chondrocyte implantation
CT: computer tomography
FeCA: femoral cartilage surface area
GRAPPA: generalized autocalibrating partially parallel acquisition imaging
ICC: interclass correlation coefficient
MACI: matrix‐induced autologous chondrocyte implantation
MRI: magnet resonance imaging
PCA: patella cartilage surface area
Px: pixels
SCDS: surface corrected defect size
TEA: transepicondylar distance
TSE: turbo spin‐echo

## INTRODUCTION

Acute injuries, repetitive overload and degenerative processes often lead to cartilage defects or degeneration in the knee joint, often leading to knee pain [6, 33]. As cartilage is avascular, spontaneous healing often fails to take place [1, 15]. Untreated cartilage defects can eventually lead to knee joint osteoarthritis.

Fortunately, there are therapeutic options to facilitate cartilage regeneration. One of the most common regenerative therapies is microfracturing, a technique popularized by Steadman [1, 15, 27]. This results in the leakage of stem cell‐rich blood from small holes placed in the subchondral bone with a dedicated instrument. The resulting blood clot transforms into fibrous cartilage. This procedure can be combined with biomaterial via matrix‐augmented bone marrow stimulation. Autologous Chondrocyte implantation, as a two‐step procedure, is suitable for defect sizes exceeding 2.5 cm^2^ [1, 4, 6, 15, 17, 27]. One‐step procedures are also available, such as osteochondral allogenic or autologous transplantation [2, 34] and the minced cartilage procedure [5]. Histologically and molecularly speaking, the results from osteochondral transplantation and ACI are believed to most closely resemble native cartilage [3, 7, 26, 30].

The choice of cartilage regeneration procedure largely depends on the size of the defect [17, 18]. despite the fact that there is little correlation between defect size and clinical outcome [11, 19]. This might be due to the fact that other parameters such as availability, defect depth and subchondral bone must also be considered [15, 17]. This fails to take into account that the femoral condyle's total cartilage surface area varies from individual to individual.

To perform targeted, individualized regenerative cartilage therapy, it is considered important to understand the relationship between the cartilage defect size and the total cartilage surface area.

The percentage of surface area that the cartilage defect covers is not being considered in current clinical therapy decisions and plays no role in current scientific analyses of regenerative cartilage therapy because of the complexity of measuring the total cartilage surface [19, 21].

The simple reason for this is that three‐dimensional parameters are technically so difficult to determine. It would therefore be advantageous to have a two‐dimensional parameter to determine which could then help us estimate a three‐dimensional structure.

The hypothesis of this study is that the transepicondylar distance (TEA) could be utilized as a parameter for assessing the total cartilage area of the femur.

## MATERIALS AND METHODS

This study was approved by the Institutional Review Board (Freiburg University's Ethics Committee approved this study, ID 443/16), and all the subjects provided written informed consent before participation. The subjects took part voluntarily in the study in accordance with the Declaration of Helsinki.

This study was registered in the Clinical Trials Register (DRKS 00011408).

**Figure 1:**
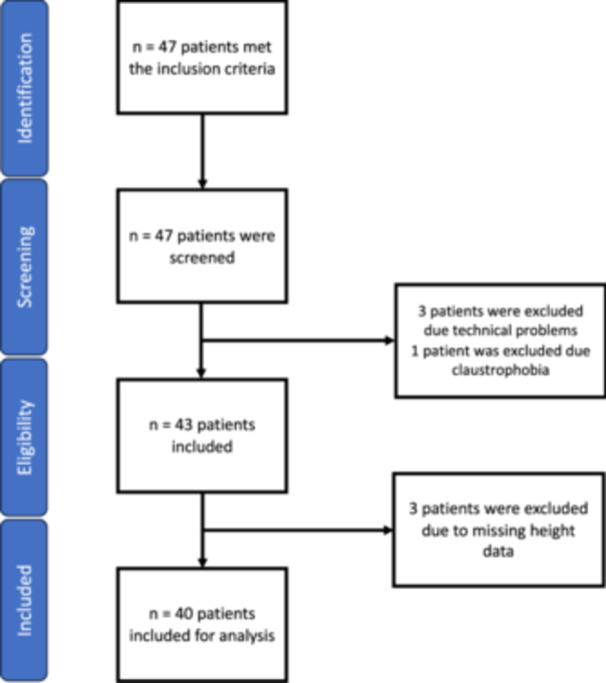
Patient selection process according to STROBE guideline.

A total of 47 subjects were initially screened and deemed eligible for inclusion in this study. One subject was excluded due to claustrophobia that became apparent during the study. Two additional subjects were excluded for technical reasons. Another three subjects were excluded from data analysis due to missing height data. A total of 40 data sets were evaluated and analysed (Figure 1).

Anatomic parameters at the distal femur and patella were analysed in this prospective cohort study. These three‐dimensional parameters were correlated with the two‐dimensional parameters.

Subjects were recruited through orthopaedic outpatient clinic and private contacts.

Claustrophobia, pregnancy, preoperated knee joints and tattoos were exclusion criteria.

### Population group

Parameters for the 40 subjects are as follows: age (18−64 years, mean 30.1 ± 8.7), gender (24 females, 16 males), height (160−196 cm, mean 176.2 ± 8.8 cm), weight (54−137 kg, mean 73.5 ± 14.5 kg) and body mass index (17.6–40.0 kg/m2, mean 23.8 ± 4.0 kg/m2).

Healthy volunteers were recruited to investigate and establish segmentation models of the femur and patella, including their cartilage and the menisci and ligaments of the knee joint. During recruitment, volunteers were selected to ensure a large range of heights, thus making the segmentation robust and generally applicable.

### MRI analysis

MRI scans were performed on a Magnetom Trio 3 T System (Siemens Healthineers). An 8‐channel multipurpose coil (NORAS MRI products) was used for signal reception. A 3D turbo‐spin echo (TSE) protocol with GRAPPA parallel imaging acceleration by a factor of 2, and isotropic resolution of 0.5 mm was applied for the MRI scans. Further scan parameters were: repetition time = 1.8 s, echo time = 59 ms, receiver bandwidth = 504 Hz/Px and scan duration = 6:20 min.

These data were postprocessed and quantified using the browser‐based, customizable SATORI platform developed by Fraunhofer MEVIS. This graphic annotation and frontend analysis is based on the MeVisLab rapid prototyping environment for medical image analysis and visualization. Segmentation was performed slice‐by‐slice, semiautomatically assisted, by an orthopaedic surgeon.

The distal femur and patella bones were automatically segmented by the software. Measurements were adjusted manually when necessary. The cartilage was then delineated. After the cartilage and bone dimensions were determined, the surface of the cartilage was identified. The software calculated both the femoral cartilage surface area (FeCA) and patella cartilage surface area (PCA). Additionally, the distance between the bony epicondyles was defined as the epicondylar distance (TEA). The maximum length of the patella in craniocaudal direction was defined as the patella length.

**Figure 2:**
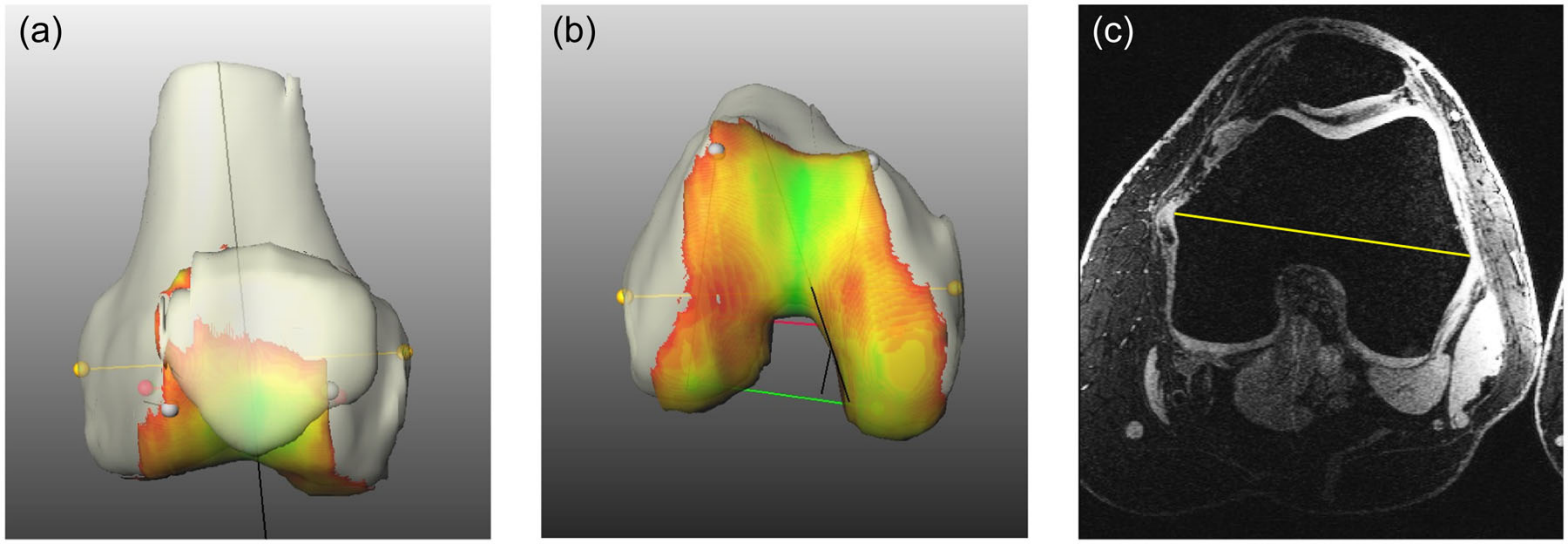
Three‐dimensional reconstruction of a right femur. The bone is beige, thinner cartilage is red, and thick cartilage is green. The yellow line marks the transepicondylar distance. (a) Anterior‐posterior view of the distal femur and patella. (b) Inferior view of the femur cartilage area. (c) Axial MRI view of the distal femur for manually measuring the TEA distance (yellow line).

The TEA distance was also measured by a resident and a senior physician in orthopaedics at two separate timepoints (Figure 2).

To prevent any potential bias, subjects were randomly selected. The MRIs were evaluated in the same manner for all subjects with the examiners unaware of their TEA distance or body size.

### Statistical analysis

Data analyses were performed by the IBM® SPSS® Statistics Version 28 for Mac. Correlation analyses were performed on MRI parameters and the different cartilage areas. No significant outliers were found, and the data was determined to be metric and to display a linear relationship. Moreover, a bivariate uniform distribution was observed, allowing the calculation of the Pearson correlation, which was categorized as poor (*r* < 0.21), fair (*r* = 0.21−0.4), moderate (*r* = 0.41−0.6), good (*r* = 0.61−0.8) or excellent (*r* = 0.81−1.0) [31].

Linear regression analyses were defined as *R* ^2^ > 0.6 good, *R* ^2^ > 0.4 moderate and *R* ^2^ < 0.4 as poor [8].

The interclass correlation coefficient (ICC) was used to determine intra‐ and interobserver reliability between the two investigators and the computerized evaluation. An ICC of <0.5 was defined as poor, 0.51−0.75 moderate, 0.76−0.9 good and >0.91 as excellent [12].

A power analysis was conducted using G*Power (Version 3.1.9.6) to determine the minimum sample size required for the Pearson's correlation. This analysis relied on an estimated power of 0.95, an *α* of 0.05 and a correlation p H1 of 0.5. This resulted in a calculated minimum sample size of 38 patients.

## RESULTS

### Anatomical parameters

During the three‐dimensional assessment of the different anatomical parameters, a total cartilage area surface of the distal femur of 72.7 cm² (±15.5 cm²) was noted. The patella's mean cartilage area surface measured 16.2 cm^2^ (±3.7 cm^2^).

Based on the three‐dimensional data set, parameters available in regular MRI knee examination sequences were also measured. TEA has a mean value of 79.1 mm (±7.7 mm). The cranio‐caudal extent of the patella was found to measure 41.1 cm (±4.0 mm).

### Intra‐ and interobserver correlation

The TEA distance was measured by two independent investigators. This value was also calculated by the computer programme. As the computer always calculates the same TEA distance from the data set, it reveals excellent intraobserver reliability. However, both iInvestigator 1 and investigator 2 also showed excellent values of 0.99 (*p* < 0.001) when calculating the interobserver correlation coefficient. Good agreement with a coefficient of 0.99 (*p* < 0.001) was also shown by the two examiners' interobserver reliability. The ratings between the two examiners and the computer rating were analysed, demonstrating high reliability with an interobserver correlation coefficient of 0.97 (*p* < 0.001).

### Correlation analysis

Body height (176.2 ± 8.8 cm), being an easily measurable individual parameter, reveals a good correlation (*r *= 0.722, *p* < 0.001) with the distal femur's cartilage area surface (FeCa) (72.7 cm^2^ ± 15.5 cm^2^). However, regression analysis shows only moderate dependence (*R* ^2^: 0.514, *p* < 0.001).

An excellent correlation was identified (*r* = 0.830, *p* < 0.001) between the TEA distance (79.1 mm ± 7.7 mm), an easily measurable two‐dimensional MRI parameter and the distal femur's total cartilage area surface (FeCA) (72.7 cm^2^ ± 15.5 cm^2^). Regression analysis yielded a good value here (*R* ^2^: 0.684, *p* < 0.001). The TEA distance therefore has higher predictive value in relation to the cartilage surface of the distal femur than to body height.

**Figure 3:**
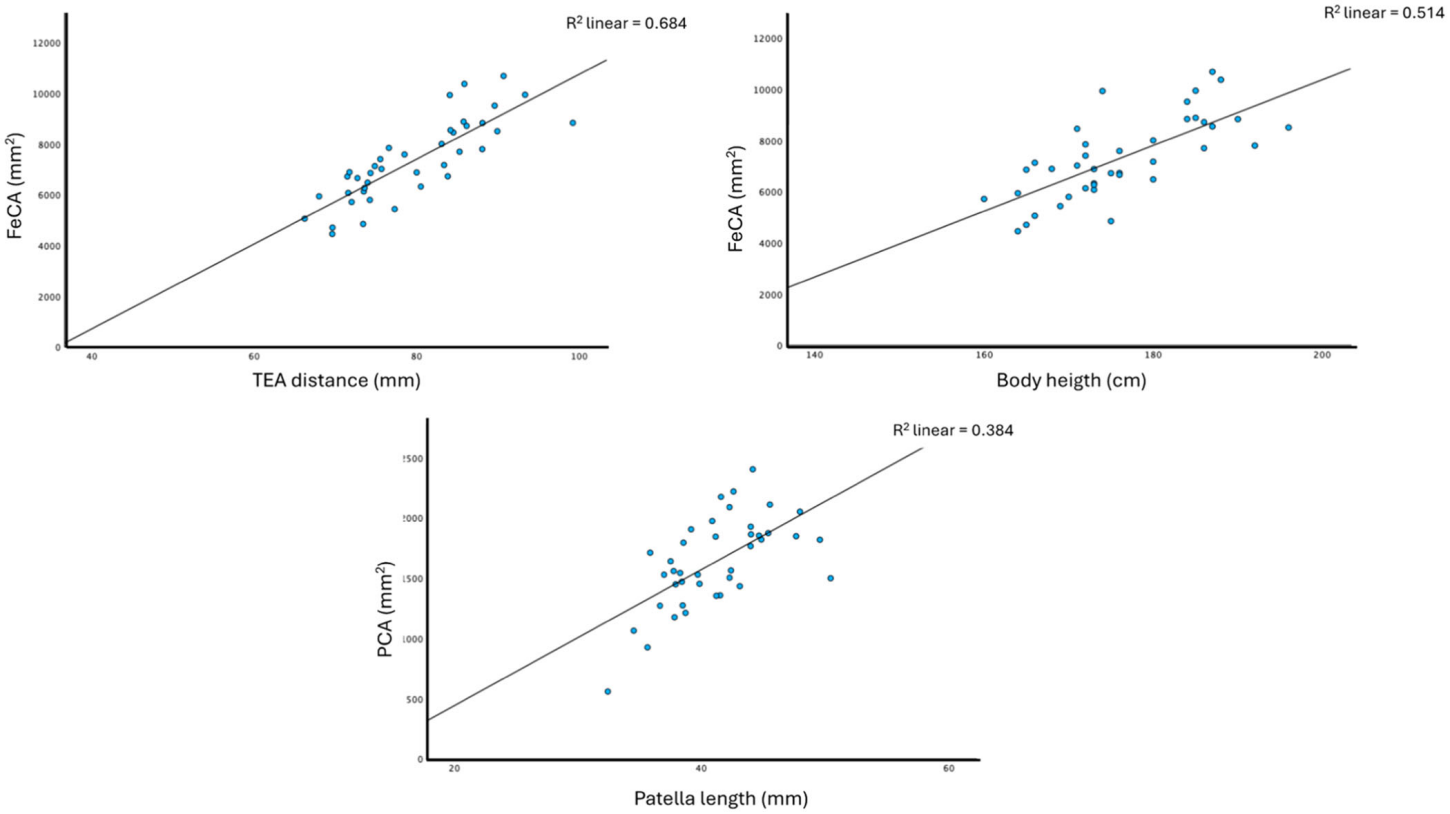
Linear regression analyses of the three‐dimensional parameters patellar cartilage area (PCA) and femur cartilage area (FeCA), compared to the one‐dimensional parameters (body height, transepicondylar distance (TEA) and patella height). Only the TEA and the femur cartilage area show good linear regression.

The patella's cranio‐caudal length (41.1 cm ± 4.0 mm) was chosen as an easily measurable two‐dimensional parameter to identify any correlation with the total cartilage surface area of the patella (16.2 cm^²^ ± 3.7 cm^²^). These results yielded a poor correlation (*r* = 0.577, *p* < 0.001) between parameters. The regression analysis also shows a poor value (*R* ^2^: 0.384, <0.001). Nevertheless, these results are significant (Figure 3) (Table 1).

**Table 1:** The table shows the correlation analysis data of various parameters.

Compared parameters	Correlation (*r*)	*p* Value
TEA dDistance (mm)/femur cartilage area surface (mm^2^)	0.830	<0.001
Body height (cm)/femur cartilage area surface (mm^2^)	0.722	<0.001
Patella length (mm)/patella cartilage area surface (mm^2^)	0.577	<0.001

*Note*: The TEA distance shows an excellent and significant correlation with the femur cartilage area surface of the distal femur. Body height shows a good, significant correlation with the femur cartilage area surface. Patella height in relation to the patella cartilage area surface seems to be poor.

## DISCUSSION

The most important finding of the present study was that, due to the difficulty to determine or estimate the total cartilage surface in clinical routine and scientific procedures, the TEA was identified as a good and reliable parameter for this purpose. This finding opens up new possibilities in the clinical therapy of cartilage lesions and in the scientific evaluation of data from patients undergoing cartilage regenerative therapy.

The size of a cartilage defect can now be evaluated as an absolute total size, but now also as a cartilage defect size standing in relation to the total cartilage surface.

There is a great need among cartilage surgeons for better methods to determine the size of cartilage surfaces and cartilage defects. Before surgery, the size of a cartilage defect can be accurately measured by optimizing the interpretation of MRI data [22]. Even if measurement techniques estimating the size of a cartilage defect during arthroscopy of the knee have been developed from the simple scaled hook to a computer‐based measurement [16, 24, 25], the total cartilage area of the femur is still not known in clinical routine. A supposedly small cartilage defect could be a relatively large one if the patient has little total femur cartilage. This simple factor could affect the clinical outcome after a cartilage repair procedure on the knee.

The TEA distance can serve as a parameter by which to ascertain the femur's total cartilage area via a calculation formula. A short TEA distance indicates that the patient has a smaller cartilage surface area. This information may then be used to determine the surface corrected defect size (SCDS). Further investigations are required to clarify the clinical relevance of SCDS, which will be easily determined by relying on this derived parameter.

Previous studies have already shown that the body height [20, 21] or tibial length suffice for a rough estimation of the cartilage surface [29]. The results of those studies [20, 21] are comparable to this study, with a moderate regression of body height to total cartilage area. It is indicated by these findings that, although body height is a useful parameter, the TEA distance is exhibited as having a highly significant correlation and good regression, making it a more accurate and reliable indicator of the distal femur's total cartilage area. A good regression results in better reproducibility of calculated values. It is therefore maintained that the TEA distance is the optimal parameter for calculating the femoral cartilage surface.

There is ample evidence of the reliable accessibility of the TEA distance in axial slices. Even when endoprostheses were implanted that led to artifacts, different observers proved able to acquire good, reproducible results [9, 14, 28]. As the bony structure is well mapped during MRI [23, 32], CT measurements can be adapted to MRI. This study also conclusively demonstrates the TEA's excellent reproducibility when measured via MRI.

In this study, it has been demonstrated that the TEA distance is a reliable parameter measurable during MRI. In turn, strong evidence of a reliable and new parameter for determining the femoral total cartilage surface area at the knee joint is delivered in this study. This will therefore enable the inclusion of the SCDS in clinical routines as well as in research.

There is no clear correlation between clinical outcomes after cartilage surgery and the absolute defect size [11, 19]. Although it is theoretically conceivable that they may be interrelated, parameters such as diffusion distance [10] and mechanical stability could play a role in various cartilage regenerative procedures [13], the absolute defect size parameter may also be unsuitable, as the relation between the cartilage defect and total surface area may be what is decisive.

Statistically significant results were obtained in the cohort of this study. Although a relatively small study group was used, it is just above the minimum number required by the power analysis. Assessing larger populations could also yield significant results for the patella, as outliers also make more of a difference in a smaller area.

Manual measurement of the TEA distance (theoretically during routine MRIs) may reveal stronger differences to computer‐aided three‐dimensional evaluations, as axial slices cannot always be positioned identically, since MRIs are usually performed by various staff using different equipment. Under study conditions, it is easier to achieve accuracy when aligning the sequences on the same device, usually by the same employee. This is probably why such excellent agreement between the computer‐assisted and manual measurements was observed in this study. An internal case series verified that there were no relevant deviations from manually measured TEA distance in routine MRIs.

The TEA distance can be easily measured in axial MRI slices of the knee joint in clinical routine. This in turn would enable the treating orthopaedic surgeon to estimate the cartilage surface in clinical routine with no significant additional effort, for example to determine the relative size of the cartilage defect (surface‐corrected defect size).

## CONCLUSION

The TEA is a reliable parameter for estimating the cartilage area of the femur using MRI. The simplicity of determining this parameter enables the femur's total cartilage area to be estimated, thus allowing an estimation of the SCDS in daily routine without requiring significant additional effort from the treating orthopedic surgeon.

## AUTHOR CONTRIBUTIONS

Tayfun Yilmaz and Kaywan Izadpanah designed the study and collected data. Tayfun Yilmaz performed the statistical analysis. Tayfun Yilmaz wrote the manuscript. Tayfun Yilmaz, Markus Siegel and Elham Taghizadeh carried out the measurements. Markus Siegel, Kaywan Izadpanah, Andreas Fuchs, Philipp Niemeyer and Hagen Schmal helped with data interpretation and critically reviewed the manuscript. All authors read and approved the final manuscript.

## CONFLICTS OF INTEREST STATEMENT

Support was received from Deutsche Forschungsgemeinschaft (grants IZ 70/2‐), the National Institutes of Health (grant 13GW0277) and the Oskar–Helene–Heim Foundation.

## ETHICS STATEMENT

This study was approved by the Institutional Review Board (Freiburg University's Ethics Committee approved this study, ID 443/16) and all the subjects provided written informed consent before participation. The subjects voluntarily took part in the study in accordance with the Declaration of Helsinki. The study was registered in the Clinical Trials Register (DRKS 00011408).

## Data Availability

All relevant data are provided within the manuscript. The data sets used and/or analysed during the current study are available from the corresponding author on reasonable request.
